# Supplementary material for: Immune Gene Repertoire of Soft Scale Insects (Hemiptera: Coccidae)
Source: Int J Mol Sci. 2024 Apr 30;25(9):4922. doi: 10.3390/ijms25094922 (PMC11084805; doi:10.3390/ijms25094922)
Supplement: Supplementary file 1 [file ijms-25-04922-s001.zip › ijms-2965955-supplementary.pdf]

Table S1. Antimicrobial peptides from Hemiptera species used to query *Ericerus pela* genome.

Organism	NCBI protein ID	Gene name	*D. melanogaster* NCBI protein ID	*A. pisum* NCBI protein ID	Best matches (e-value)
Cicada flammata	P83282.1	cicadin	not found	not found	not found
Cryptotympana dubia	P85028.1	cryptonin	not found	not found	not found
Palomena prasina	P80411.1	metalnikowin-3	not found	not found	not found
Peregrinus maidis	WMM65954.1	lugensin	not found	not found	not found
Plautia stali	BBE08155.1	hemiptericin	not found	not found	not found
	BDD37282.1	pentatomicin	not found	not found	not found
Podisus maculiventris	AAB36066.1	thanatin	not found	not found	not found
Pyrrhocoris apterus	4EZN_D	pyrrhocoricin	not found	not found	not found
